# Dihydroartemisinin induces autophagy-dependent death in human tongue squamous cell carcinoma cells through DNA double-strand break-mediated oxidative stress

**DOI:** 10.18632/oncotarget.17520

**Published:** 2017-04-29

**Authors:** Xinli Shi, Li Wang, Xiaoming Li, Jing Bai, Jianchun Li, Shenghao Li, Zeming Wang, Mingrui Zhou

**Affiliations:** ^1^ Department of Otolaryngology Head and Neck Surgery, Bethune International Peace Hospital, Shijiazhuang 050081, China; ^2^ Department of Pathobiology and Immunology, Hebei University of Chinese Medicine, Shijiazhuang 050200, China; ^3^ Laboratory of Organ Fibrosis Prophylaxis and Treatment by Combine Traditional Chinese and Western Medicine, Research Center of Combine Traditional Chinese and Western Medicine, Affiliated Traditional Medicine Hospital of Southwest Medical University, Luzhou 646000, China

**Keywords:** dihydroartemisinin, human tongue squamous cell carcinoma, autophagy, DNA double-strand break, STAT3

## Abstract

Dihydroartemisinin is an effective antimalarial agent with multiple biological activities. In the present investigation, we elucidated its therapeutic potential and working mechanism on human tongue squamous cell carcinoma (TSCC). It was demonstrated that dihydroartemisinin could significantly inhibit cell growth in a dose- and time-dependent manner by the Cell Counting Kit-8 and colony formation assay *in vitro*. Meanwhile, autophagy was promoted in the Cal-27 cells treated by dihydroartemisinin, evidenced by increased LC3B-II level, increased autophagosome formation, and increased Beclin-1 level compared to dihydroartemisinin-untreated cells. Importantly, dihydroartemisinin caused DNA double-strand break with simultaneously increased γH2AX foci and oxidative stress; this inhibited the nuclear localization of phosphorylated signal transducer and activator of transcription 3 (p-STAT3), finally leading to autophagic cell death. Furthermore, the antitumor effect of dihydroartemisinin-monotherapy was confirmed with a mouse xenograft model, and no kidney injury associated with toxic effect was observed after intraperitoneal injection with dihydroartemisinin for 3 weeks *in vivo*. In the present study, it was revealed that dihydroartemisinin-induced DNA double-strand break promoted oxidative stress, which decreased p-STAT3 (Tyr705) nuclear localization, and successively increased autophagic cell death in the Cal-27 cells. Thus, dihydroartemisinin alone may represent an effective and safe therapeutic agent for human TSCC.

## INTRODUCTION

As the number one killer and the most common epithelial cancer identified in the oral cavity, tongue squamous cell carcinoma (TSCC) has the major characteristics of high lymph node metastasis, regional recurrence rate and resultant dissatisfactory treatment outcome [1]. The poor prognosis of the disease and the major side effects of the available pharmacological treatments create an urgent need for de novo chemotherapeutic strategies and drugs.

As the active ingredient of *Artemisia annua* L., Artemisinin has been developed as anti-malarial drug and used worldwide [2]. Interestingly, dihydroartimisinin (DHA), an FDA-approved artemisinin-derived antimalarial drug, significantly inhibits cancer cell growth *in vitro* and *in vivo* [3]. Therefore, artemisinin-type drugs are at the stairways to the clinics. However, there are only limited experimental studies examining the potential of DHA for treating TSCC.

It is known that artemisinin eliminates plasmodium parasites through the induction of iron-dependent oxidative stress [4]. Thus, DHA might be an effective anti-cancer chemotherapeutic drug by regulating redox homeostasis [5, 6]. Signal transducers and activators of transcription 3 (STAT3) plays a key role in oxidative stress-mediated tissue injury [7]. Currently, we have confirmed that DHA is a putative STAT3 inhibitor and induces apoptosis by Jak2/STAT3 pathway in head and neck squamous carcinoma cells [8]. Macroautophagy (autophagy) is a stress-responsive and homeostatic mechanism for clearance damaged cellular components. Physiologically, autophagy maintains viability and homeostasis through a lysosomal degradation pathway in normal cells. However, it also triggers the death of cancer cells under certain circumstances [9]. Consistently, some studies suggested that DHA showed anti-tumor effect via autophagy on glioma cells [10], cisplatin-resistant ovarian cancer cells [11], esophageal cancer cells [12], pancreatic cancer cells [13], and human myeloid leukemia K562 cells [14].

Recently, different subcellular localization patterns of STAT3 affect autophagy in various ways [15]. For example, cytoplasmic STAT3 acts as a tonic inhibitor of autophagy, and nuclear phosphorylated STAT3(Tyr705) tightly regulates autophagy via the transcriptional regulation of several autophagy-related genes such as *BECN1* [16]. In baseline conditions, STAT3 mainly exists in the cytoplasm, transcriptionally inactive monomers or dimers. Once phosphorylated on tyrosine and serine residues, dimers get stabilized and enter into the nucleus. Here, we reported that DHA significantly inhibited the growth in human TSCC Cal-27 cells *in vitro* and *in vivo*, which may be attributed to enhanced autophagic cell death through decreased phosphorylated STAT3(Tyr705) nuclear localization mediated by genomic and oxidative stress. Therefore, we provided further solid experimental evidence to support DHA as a potential therapeutic agent for human TSCC.

## RESULTS

### The inhibition of Cal-27 cells proliferation *in vitro* by DHA

**Figure 1:**
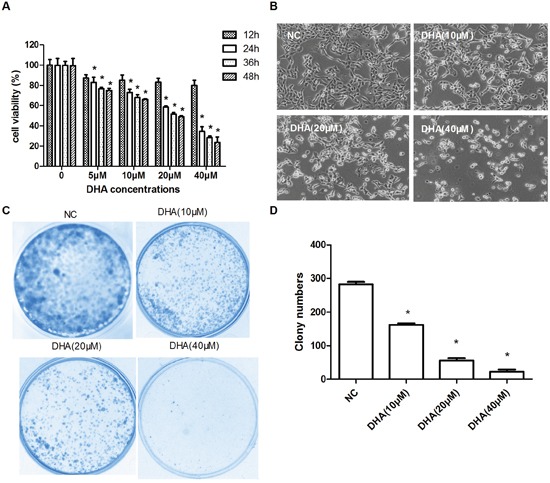
The inhibition of Cal-27 cells proliferation *in vitro* by DHA **(A)** CCK8 to test the inhibitory effect of DHA on Cal-27 cell proliferation. Cal-27 cells were treated with DHA as indicated for different times (mean ± SD, n=3). *P<0.05 vs. control. **(B)** The decreased cell number was detected at 24h treatment with different concentrations of DHA (100×). **(C)** Representative photographs of clonogenic assay. All experiments were performed at least for three times. **(D)** Statistical analysis of the number of colony at 24 h. The inhibition effect on growth and proliferation was calculated by number of formed cell clones. Data are shown as the mean ± SD (n = 3). *P<0.05 vs. NC group.

DHA is selectively cytotoxic to some cancer cell lines [3]. To test the anti-proliferative effect of DHA *in vitro*, human TSCC Cal-27 cells were respectively exposed to DHA (5, 10, 20 and 40 μM) for 12, 24, 36 and 48 h. After this treatment, cell proliferation and cytotoxicity assay (CCK-8) was conducted to assess cell viability. It was shown that DHA with greater concentrations inhibited the growth of Cal-27 cells more significantly, and its inhibition rate also increased as time went on (Figure 1A). The result suggested that DHA cytotoxicity was dose- and time-dependent. However, DHA showed less inhibitory effect at 12 h compared to that at any other separate time points (Figure 1A). Hence, 24 h was chosen as the treatment time. Next, a clonoy assay was performed with DHA (10, 20 and 40 μM, respectively) for 24 h to determine whether DHA affected longterm colony formation. As expected, we observed that the quantity of DHA-treated cells decreased in greater concentration of DHA at 24 h (Figure 1B), with a 50% inhibiting concentration (IC50) of 24.35 μM. Meanwhile, the surviving colonies were also markedly inhibited as per clone formation test (Figure 1C and 1D). Taken together, these results suggested that DHA inhibited the growth and proliferation of Cal-27 cells *in vitro* in both dose- and time-dependent manners.

### The induction of autophagy in Cal-27 cells by DHA

**Figure 2:**
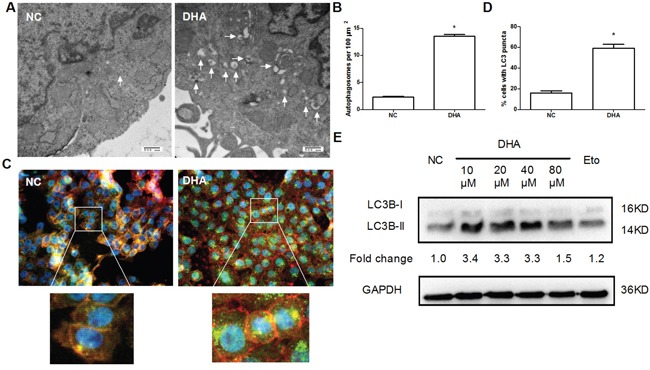
The induction of autophagy by DHA in Cal-27 cells **(A)** Autophagosomes were checked by transmission electron microscopy. Cal-27 cells were treated with or without 24.5μM DHA for 24h, then fixed, embedded, sectioned, mounted, and stained as described in Materials and Methods. The autophagosome was indicated by white arrow. Scale bar 500 μm. **(B)** The quantitative data for autophagosomes were obtained from 3 sets of experiments. A total of 20 cells were counted for each variable. * *P*<0.05 vs. NC group. **(C)** DHA-induced autophagosomes were detected in Cal-27 cells by immunofluorencent staining of LC3B (green) (1000×). Cells were treated as described above. F-actin (red) was stained with Phalloidine. Nuclei (blue) were counter-stained with DAPI. **(D)** Quantitative analysis of autophagosomes with green-fluorescent puncta. Fifty cells were counted for each variable. * *P*<0.05 vs. NC group. **(E)** The autophagy-associated proteins LC3B-I/II were detected by Western blotting. Cal-27 cells were treated with indicated concentrations of DHA for 24 h, and then harvested for examining the expression levels. 40μM Etopside was used as DNA double-strand break positive control. GAPDH was used as a loading control. All experiments were performed in triplicates.

Through the above experiment, it was known that DHA inhibited the growth and proliferation of Cal-27 cells *in vitro*. But what was the mechanism behind it? As is known, autophagy is a common event in cancer chemotherapy [17]. First of all, we thought whether DHA induced autophagy? Thus, we performed an ultrastructural analysis to morphologically demonstrate the presence of autophagy in DHA-treated cells. Morphological changes were observed by transmission electron microscopy. As expected, it was noted that the quantities of autophagosomes, double and multiple membrane-encapsulated components were higher in the DHA-treated group than those in the NC group (Figure 2A and 2B). The result suggested that DHA induced autophagy in Cal-27 cells.

Light chain 3B (LC3B) is the widely accepted marker for the assessment of autophagy activity [18]. The formation of autophagosome is associated with the conversion of LC3B from LC3B-I to LC3B-II. To further confirm the effect of DHA on the autophagy in Cal-27 cells, autophagosomes were observed by immunofluorescent staining with LC3B antibody. Autophagosomes were presented as green fluorescent puncta under fluorescence microscopy. Accordingly, we found that DHA-treated cells showed bright green fluorescence (Figure 2C) and increased autophagosomes (Figure 2D). The result suggested that DHA can promote the formation of autophagosome in Cal-27 cells. Furthermore, Western blot analysis showed that DHA (10, 20 and 40 μM, respectively) promoted the conversion of LC3B-I to LC3B-II and increased the expression level of LC3B-II (Figure 2E). Overall, these results demonstrated that DHA treatment of Cal-27 cells could promote autophagy, as is evidenced by increased autophagosome formation and increased LC3B-II level, and increased conversion of LC3B-I to LC3B-II, compared to NC group. Meanwhile, the effect of DHA on autophagy at each concentration was similar to that of Etoposide (40μM), an agent capable of inducing DNA double-strand break (Figure 2E).

### The induction of nuclear DNA double-strand break in Cal-27 cells by DHA

**Figure 3:**
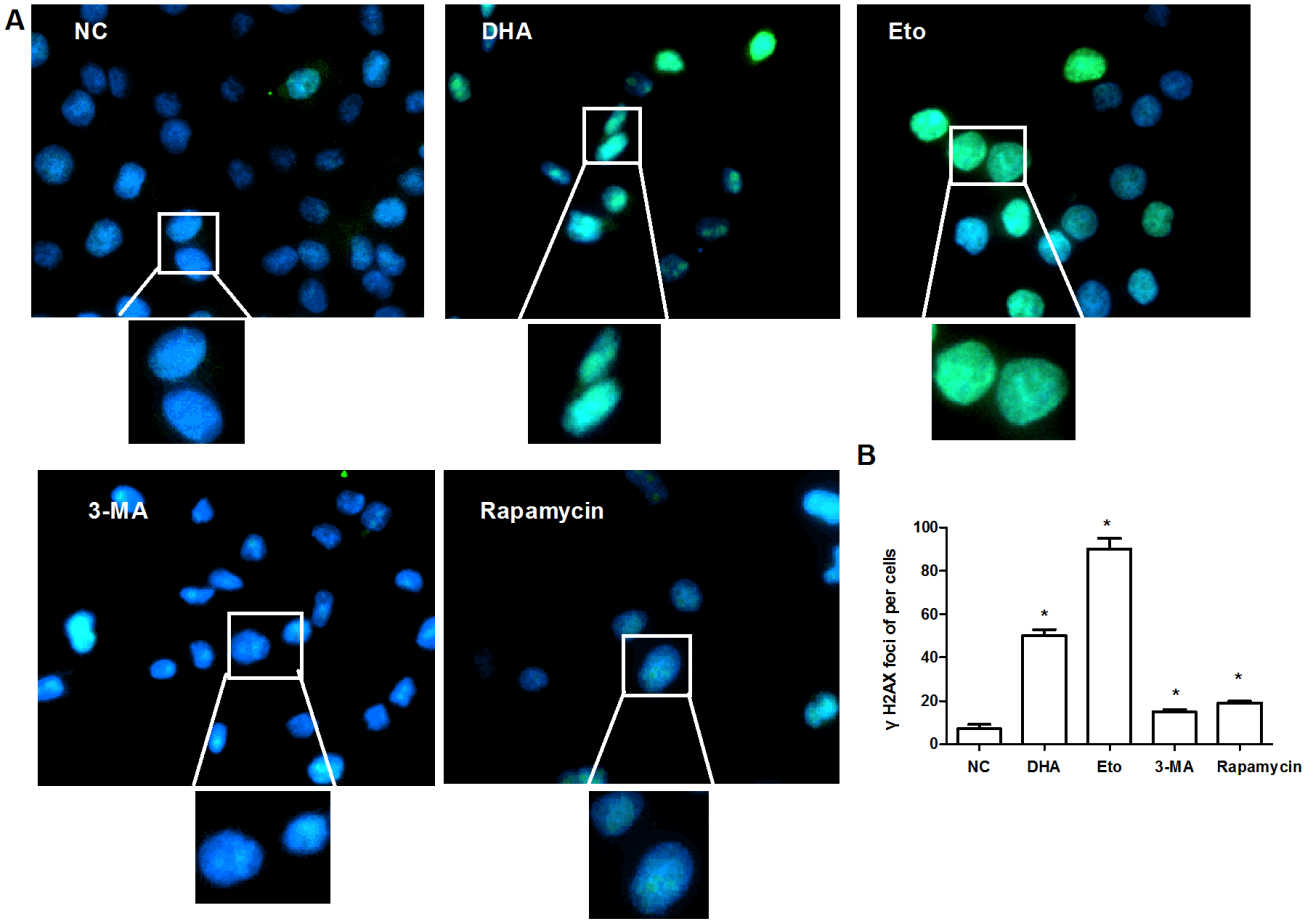
The induction of nuclear DNA double-strand break by DHA in Cal-27 cells **(A)** Representative images of γH2AX foci formation. Cal-27 cells were treated with 0.1% DMSO or DHA (24.5 μM) for 24 h, and analyzed for γH2AX (green). Etopside (40μM) was used as DNA DSB positive control. 3-MA (1 mM) and rapamycin (0.1 μM) acted as autophagy inhibitor and activator, respectively. Nuclei were counter-stained with DAPI (blue). The upper and bottom panels are respectively 400× and 1000×. **(B)** Statistical analysis of the number of γH2AX foci. Data are shown as the mean ± SD (n = 3). * *P* < 0.05 vs. NC group.

DNA double-strand break is one of the most critical DNA lesions related to cell-death and genomic integrity. We hypothesized that DHA might induce double-strand break to trigger autophagy in Cal-27 cells. The phosphorylation of H2AX at Ser139, γH2AX, is an early marker for the identification of DNA double-strand break [19]. In fact, γH2AX foci on chromosomes represent repaired lesions or unrepaired DNA breaks [20]. To observe DHA-induced γH2AX foci formation, Cal-27 cells was treated with 24.5 μM DHA (IC50=24.35 μM) for 24 h. As expected, DHA significantly increased the counts of γH2AX foci, with comparable effects as the Etoposide treatment, the DNA double-strand break positive control [21] (Figure 3A and 3B). The result suggested that DHA can damage DNA via activating H2AX-mediated double-strand break in Cal-27 cells.

As one of the most widely used inhibitor of phosphoinositide 3-kinase (PI3K), 3-MA inhibits autophagy by blocking the activity of the Beclin-1-PI3K complex. Meanwhile, Rapamycin is an mTOR inhibitor that up-regulates autophagic activity. To investigate the effect of autophagy on DNA double-strand break, we blocked autophagy with 3-MA (1 mM) and promoted autophagy activity with rapamycin (0.1 μM) [22], and happened to find that the formation of γ-H2AX foci was prolonged in both treatments (Figure 3A and 3B). Collectively, autophagy is the downstream event of the double-strand break caused by DHA.

### The increase of oxidative stress in Cal-27 cells by DHA-mediated DNA double-strand break

**Figure 4:**
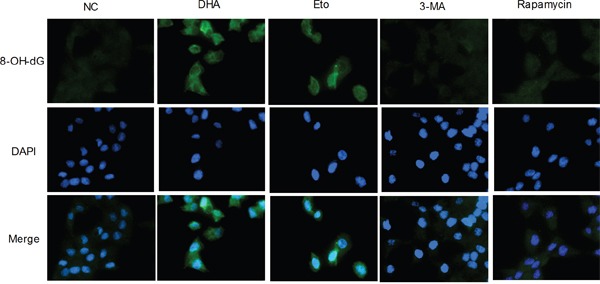
The increase of oxidative stress by DHA-mediated DNA double-strand break in Cal-27 cells Representative images of oxidative cellular damage by immunofluorescence assay (1000×). Cal-27 cells were treated as described above for 24 h and analyzed for 8-OH-dG (green). Nuclei were counter-stained with DAPI (blue).

DNA damage increases oxidative stress [6]. Mitochondrial DNA (MtDNA) is 10 to 100 times more sensitive to oxidative stress than nuclear DNA [23] and thus highly susceptible to oxidative damage. To detect whether DHA stimulated cellular oxidative DNA damage, we further performed immunofluorescence assay with 8-OH-dG, a specific oxidative DNA damage marker. As expected, the green fluorescent puncta were more apparent in the cytoplasm and nucleus of DHA-treated cells comparable to those in the Etoposide group (Figure 4). The result suggested that DHA-mediated DSB damage increased cellular oxidative stress. Meanwhile, an insignificant change in 8-OH-dG green fluorescent puncta was observed in the 3-MA or Rapamycin group (Figure 4). Collectively, DHA boosted cellular oxidative stress, which may promote autophagy in Cal-27 cells.

### The disruption of STAT3 nuclear translocation by DHA

**Figure 5:**
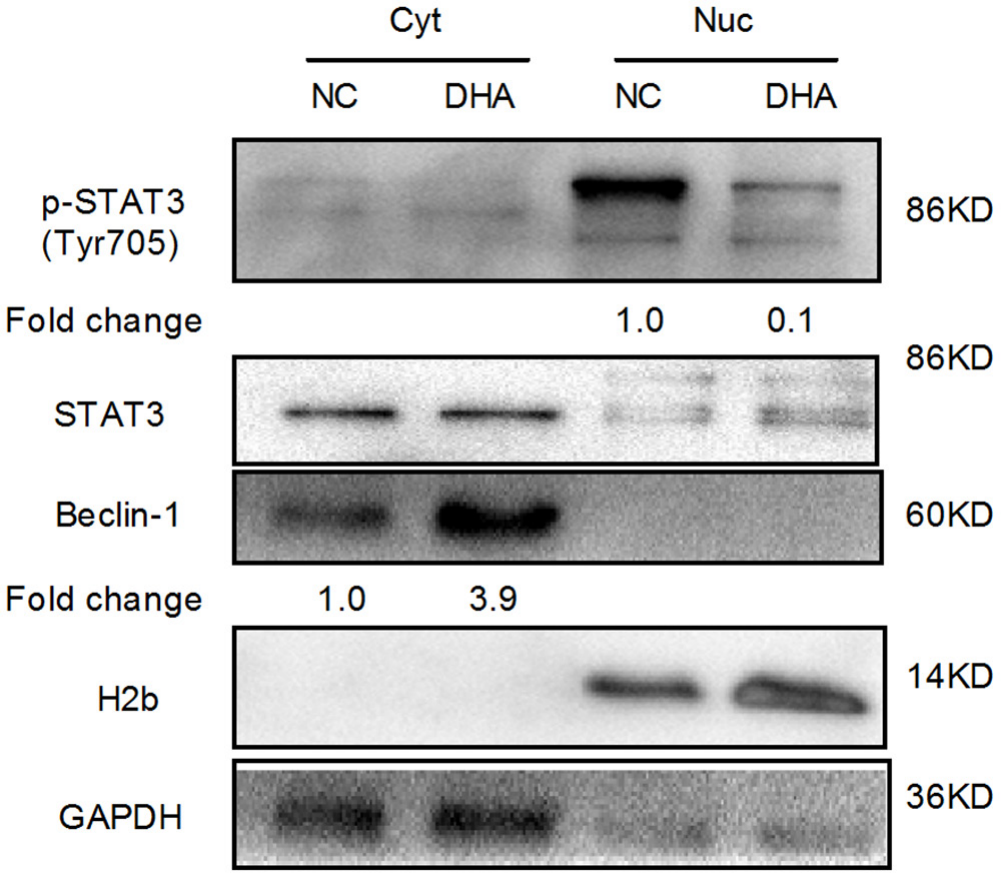
The analysis of STAT3 nuclear translocation via Western blot in Cal-27 cells H2b and GAPDH served as the quality control for the nuclear fraction and the cytoplasmic fraction, respectively. Cyt: cytoplasmic proteins; Nuc: nuclear proteins.

STAT3 acts as a stress responsive transcription factor and plays a key role in oxidative stress [16]. We have previously confirmed that DHA inhibited STAT3 activation by selective blockade of Jak2 phosphorylation in Cal-27 cells [8]. Moreover, STAT3 localization also plays an important role in autophagy [15]. Nuclear STAT3 inhibits autophagy by disrupting the formation of the BECN1/PIK3C3 complex [15]. To determine whether DHA affects the subcellular localization of STAT3, we performed Western blot analysis following the extraction of cytoplasm and nucleus. Interestingly, we detected that phosphorylated STAT3 (Tyr-705) level was decreased in the nucleus of DHA-treated Cal-27 cells compared with that in the NC group (Figure 5). Phosphorylated STAT3 (Tyr-705) is required for STAT3 dimerization and nuclear translocation. The result suggested that DHA suppressed nuclear translocation of phosphorylated STAT3 (Tyr-705). Nuclear phosphorylated STAT3 (Tyr-705) directly binds to the promoter region of BECN1 and represses its transcription. Consistent with the above result, increased Beclin-1 levels were observed in DHA-treated cells (Figure 5). Beclin-1 is essential for autophagosome formation [24]. These results suggested that decreased nuclear phosphorylated STAT3 (Tyr-705) relieved the inhibition of Beclin-1 and promoted autophagy. Additionally, cytoplasmic STAT3 fundamentally inhibited autophagy [15]. However, we demonstrated that the expression level of cytoplasmic STAT3 did not change in DHA-treated Cal-27 cells (Figure 5). Altogether, these results suggested that DHA disrupted the nuclear translocation of phosphorylated STAT3 (Tyr-705) and promoted autophagy in Cal-27 cells.

### The anti-tumor effect of DHA on Cal-27 cell xenograft tumor in mice

**Figure 6:**
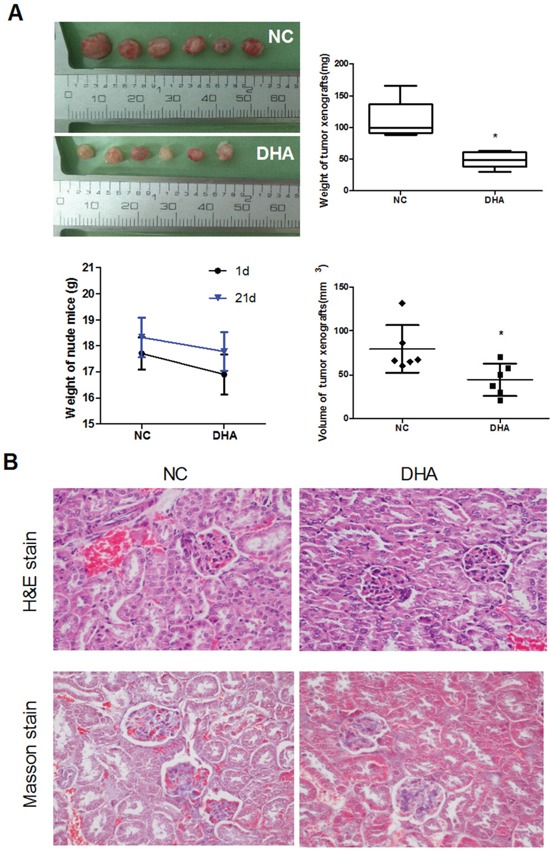
The anti-tumor effect of DHA on Cal-27 cell xenograft tumor in mice **(A)** DHA noticeably inhibited the growth of Cal-27 xenograft tumor. Nude mice were inoculated with 1×10^7^ of Cal-27 cells. After the formed tumor was palpable, mice were randomly divided into two groups. DHA (25 mg/kg) was given to the ‘DHA’ group once daily for five consecutive days per week for 21 d. Six xenografts were performed in each group. The volume and weight of tumor xenografts were presented when the mice were sacrificed. Tumor diameter was measured when the mice were sacrificed. Tumor volume was calculated by the formula: V (mm^3^) = width^2^ (mm^2^) × length (mm) ×0.5. Data are means ± SD, *P < 0.05 vs. control. The body weight changes of tumor-bearing mice at the 1^st^ and 21^th^ days. **(B)** Histological findings of the kidney were determined by H&E stain (upper panels) and Masson stain (lower panels) when the tumor-xenograft mice were sacrificed. Magnification × 400.

DHA promotes autophagy-dependent death in Cal-27 cells *in vitro*. Therefore, we further determined the anti-tumor effect of DHA in nude mice bearing Cal-27 tumor xenograft model. DHA (25 mg/kg/d) was administered by intraperitoneal injection for 21 days. The treatment effect was evaluated through the measurement of tumor volume. On average, DHA inhibited the tumor growth by 56.58% (Figure 6A). On Day 21, mice were sacrificed and the tumor weights and volumes were measured. As expected, the weight and volume of xenograft tumor were significantly reduced in DHA-treated mice (44.63±18.44mm^3^, 48.75±12.63 mg) compared with the controls (79.55±27.01 mm^3^, 112.27±29.55 mg) (Figure 6A). These results indicated that DHA noticeably inhibited the growth of Cal-27 xenograft tumor *in vivo*.

In order to investigate the potential toxic effects of DHA, especially its toxicities to the kidney, we examined the body weights, histological changes and fibrosis of the kidney in the DHA-treated group. As a result, no treatment-related changes in body weights were observed at the tested doses in DHA-treated xenograft mice (Figure 6A, lower panels). Importantly, both H&E and Masson staining images showed that no kidney injury associated with toxic effect was observed after intratumoral injection in DHA group, as is shown by abnormal tubular distortion, necrosis, and cellular debris compared to that in the NC groups (Figure 6B).

## DISCUSSION

**Figure 7:**
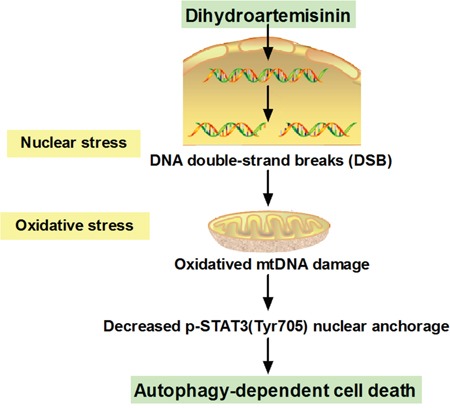
Schematic summary of the mechanism model of DHA-induced autophagic cell death in human tongue squamous cell carcinoma

DHA, the major active metabolite of artemisinin, is a powerful antimalarial medicine extracted from the Chinese herb of *Artemisia annua L* [2]. Like other natural products, DHA acts in a multi-target manner against tumors and represents an attractive candidate for cancer therapy [3]. However, the potential of DHA in treating human TSCC are just limited. In this report, we demonstrated a link between DHA, a safe and effective drug, and autophagy-related cell death through reducing phosphorylated STAT3 (Tyr705) nuclear localization in human TSCC Cal-27 cells. DHA-induced nuclear DNA double-strand break mediates cytoplasmic oxidative stress, which disrupts STAT3 nuclear translocation, and triggers autophagy (Figure 7). Meanwhile, we found that DHA alone inhibits cell growth *in vitro* and *in vivo*. Our results provided the evidence that DHA is a potential therapeutic agent for human TSCC.

In the present investigation, we demonstrated that DHA induces autophagy-related death in Cal-27 cells. Recently, the antitumor effect of DHA has been a research focus in clinical trials [25]. Consistently, some studies reported that DHA caused apoptosis in oral cancer YD-10B cells through a caspase-3-dependent pathway [26] and in head and neck cancer cells *in vitro* [8, 27]. In addition, DHA inhibited the formation of tumor in the dogs challenged with canine oral papillomavirus [28]. Furthermore, we proved that DHA-induced cytotoxic effect in Cal-27 cells was associated with autophagy, evidenced by increased autophagosome formation, increased LC3B-II level and conversion of LC3B-I to LC3B-II, and increased Beclin-1 level. Consistent with our results, one study reported that DHA stimulated autophagy by the repression of NF-κB activity in several cancer cells [29]. Indeed, some cytotoxic drugs often trigger autophagy to restore the excessive cellular damage, which promotes cell death through extensive oxidative stress [30]. Recent observations indicate that artemisinin and its derivatives resist tumor proliferation by oxidative stress [11, 31]. For instance, artesunate, a semisynthetic derivative from artemisinin, induces genotoxic stress by DNA double-strand breakin Chinese hamster ovary (CHO-9) cells [32]. Besides, it is also known that DHA can induce iron-dependent oxidative stress [12]. In the present study, we revealed that DHA induced genotoxic stress in Cal-27 cells as indicated by enhanced γH2AX expression. DHA-induced oxidation effect has been studied in some other cancers. However, DNA double-strand break by DHA has not yet to be reported in TSCC. In support of our results, another study showed that DHA increased the expression level of γH2AX and induced DNA damage response in A375 melanoma cells [31].

In the aspect of mechanism, it was found that DHA disrupted the nuclear translocation of STAT3 and increased Beclin-1 level. It is known that the Jak2/STAT3 signaling pathway plays a key role in oxidative stress [7]. Moreover, our recent studies confirmed that DHA, a putative STAT3 inhibitor, blocked Jak2 phosphorylation through Jak2/STAT3 pathway in Cal-27 cells [8]. Some studies have suggested a correlation between autophagy and the cellular localization of STAT3 in human cancer [33]. STAT3 dynamically shuttles between cytoplasm and nucleus and mainly exists in the nucleus [34]. Interestingly, we first found that DHA inhibited STAT3 nuclear translocation in Cal-27 cells. The dimer formed by phosphorylated STAT3 (Y-705) goes into the nucleus, directly binds to the promoter region of *BECN1* and represses its transcription in lung cancer cells [35]. As a core component of class III phosphatidylinositol 3-kinase complex, Beclin-1 can promote autophagy [24].

In this pathway, Beclin-1 is released from its complex with Bcl-2/Bcl-xl to initiate autophagy [36]. At the present, we determined that DHA increased the expression level of Beclin-1. Meanwhile, our previous studies showed that the expression level of Bcl-xl was reduced in DHA-treated Cal-27 cells [8]. Consistently, one clinical study showed that Beclin-1 acted as a tumor suppressor in the development or progression of TSCC [37].

In this work, we have demonstrated that DHA may serve as a potent chemotherapeutant for human TSCC. In addition, Cal-27 cells treated with DHA exhibited a lower level of DNA double-strand break compared with Etoposide *in vitro*. However, the toxic effects of DHA and the associated safety concerns in treating cancers have to be resolved before it comes into clinical use. In this regard, we established Cal-27 xenograft tumor in DHA-treated mice and found it nontoxic to kidney. Consistently, DHA-piperaquine was used in a clinical trial to treat 75 vivax malaria-infected patients, in which no patients developed acute kidney injury after 8 weeks of drug administration [38]. Moreover, a safety/efficacy study with artesunate was conducted in 23 dogs with unresectable tumors, which showed no neurological or cardiac toxicity at median dosage within 385 days and mostly transient fever and haematological/gastrointestinal toxicity in 16 dogs [39]. Furthermore, some previous reports suggested that DHA attenuate lung injury or liver fibrosis in Sprague-Dawley rats [40–42]. In addition, an observational study about the toxic effects of DHA-piperaquine on liver was conducted on 10,591 patients with uncomplicated malaria, which showed no changes in the liver enzymes after 28 days of drug application [43]. Therefore, DHA may represent a promising therapeutic agent in human TSCC with minimal or negligible toxic and side effects.

In summary, the present work demonstrated that DHA depressed the growth of cancer and human TSCC Cal-27 cells *in vitro* and *in vivo*, respectively. In the aspect of mechanism, DHA-induced DNA double-strand break enhanced oxidative stress, thereby inhibited the nuclear localization of phosphorylated STAT3 (Tyr705), and finally led to autophagy-related cell death. Furthermore, DHA did not damage the kidney in xenograft tumor-bearing mice. Our findings showed the therapeutic potential of DHA alone in human TSCC.

## MATERIALS AND METHODS

### Cell line and treatment

Human TSCC Cal-27 cells were purchased from American Type Culture Collection (Manassas, VA) and cultured in DMEM (Gibco, USA) supplemented with 10% fetal bovine serum (Gibco, USA), 100 U/ml penicillin and 100 μg/ml streptomycin at 37°C and 5% CO_2_ in an atmosphere of 100% humidity.

DHA (Tci, Japan), Etoposide (Sigma, USA), 3-MA(Sigma, USA) and Rapamycin(Sigma, USA) were dissolved in DMSO (Sigma, USA) and stored at -20°C. Cal-27 cells were treated with DHA (24.5 μM), Etoposide (40 μM), 3-MA (1 mM) or rapamycin (0.1 mM) for 24 h, respectively. The culture medium containing 0.1% DMSO was used as the control. These treated cells were subjected to transmission electron microscopy, immunofluorescence, Western blot analysis and ROS determination.

### Cell viability assay

Cal-27 cells were seeded in 96-well plates (1×10^4^ cells/well) and treated with DHA at different concentrations (5, 10, 20 and 40 μM) for 24 h. Cell viability was determined with Cell Counting Kit-8 (CCK-8, Dojindo Molecular Technology, Japan) according to the manufacturer's protocol. Finally, optical density (OD) was monitored by a Multiskan Spectrum Microplate Reader (Thermo, USA) at 450 nm, with 650 nm as the reference wavelength. The cell viability values were calculated as previously described [44]. IC50 values were obtained from the cytotoxicity curves using the SOFTmax PRO software.

### Colony formation assay

Cal-27 cells were treated with or without DHA (10, 20 and 40 μM) for 24 h. After treatment, cells were trypsinized and replated into 60 mm dishes at 600 cells per dish. After they were cultured for 14 days, the cell colonies were fixed with chilled methanol, colored by Giemsa staining, and counted under dissection microscope. Cloning with a diameter not less than 60 μm is considered a clone. Survival fraction curves were determined as previously described [45].

### Transmission electron microscopy

Cal-27 cells were treated with 24.5μM DHA for 24 h, then the treated cells were collected, and fixed with 3% glutaraldehyde, postfixed with 1% OsO4 (Sangon Biotech), dehydrated in acetone, and embedded in Epon 812 (Nissin EM, Tokyo). Ultrathin sections were stained with 2.0% uranyl acetate/lead citrate, and observed under transmission electron microscope (Hitachi, Ltd., Tokyo).

### Immunofluorescence assay

Cal-27 cells were cultured for 24 h on glass coverslips in 24-well plates (2 × 10^5^ cells/well) with or without treatment with DHA. The samples were fixed, permeabilized, blocked, and incubated with primary antibody at 37°C for 1 h and then with corresponding secondary antibody at 37°C for 1 h. The primary antibody used in this study included rabbit anti-γH2AX polyclonal antibody (bs-3185R, Bioss, diluted at 1:200), rabbit anti-LC3B antibody (#2775, CST, diluted at 1:400) and rabbit anti-8- Hydroxyguanosine (8-OH-dG) antibody (ab62623, Abcam, diluted at 1:500). The used secondary antibodies were Alexa Fluor1 488-conjugated donkey anti-rabbit IgG antibody (Invitrogen Life Technologies, 1:400) and sheep anti-rabbit Cy3-conjugated antibody (C2306, Sigma, diluted at 1:100). Cytoskeleton was stained with Phalloidine (Sigma, St Louis, MO, USA) and incubated at 37°C for 1 h. Cells were counterstained with 4′, 6-diamidino-2-phenylindole dihydrochloride (DAPI) (10μg/ml) (Sigma, USA). Images were captured via a fluorescence microscope (Olympus BX51, Japan), and assessed by confocal microscopy.

### Nuclear and cytoplasmic extraction and Western blot analysis

Cal-27 cells were seeded in 6-well plates (3 × 10^5^ cells/well), treated as described above, and fractionized to obtain the nuclear and cytoplasmic protein [46]. Whole-cell extracts were directly lysed in SDS sample buffer (50 mM Tris-HCl pH 6.8, 1% SDS, 10% glycerol, 5% β-mercaptoethan, 0.01% bromophenol blue). The primary antibodies were rabbit anti-LC3B antibody (#2775, CST, diluted at 1:400), rabbit anti-Beclin-1 antibody (#3495, CST, diluted at 1:400), rabbit anti-Phospho-STAT3 (Tyr705) antibody (#3495, CST, diluted at 1:1500), rabbit anti-STAT3 antibody (#8232, CST, diluted at 1:1000), rabbit anti-H2b antibody (BS1657, Bioworld, diluted 1:500) and rabbit anti-GAPDH polyclonal antibody (#2118, CST, diluted at 1:1000). The secondary antibody was goat anti-rabbit IgG-HRP (sc-2004, Santa Cruz Biotech, diluted 1:5000). H2b and GAPDH served as the quality controls for nuclear and cytoplasmic fractions, respectively.

### Establishment of xenograft tumors and treatment of animals

Female BALB/c nude mice (Vital River Laboratory Animal Technology Co. Ltd., Beijing) at the age of 5-6 weeks were used. Each mouse was subcutaneously inoculated with 1×10^7^ Cal-27 cells in the left inguinal area to establish the xenograft tumor. When the average tumor size reached 5 mm in diameter, the tumor-bearing mice were randomly distributed to four different groups with six animals in each group. The mice in DHA group received intraperitoneal injection of DHA in DMSO (25 mg/kg), once daily for five consecutive days per week for 21 d. The mice in normal control (NC) group were intraperitoneally injected with 0.1% DMSO in physiological saline. Tumor size and body weight were measured in each animal every 5 days throughout the study. Tumor volume was calculated by the formula: V (mm^3^) = width^2^ (mm^2^) × length (mm) ×0.5. The inhibition rate of tumor growth was calculated by the formula (1-the average tumor weight of the experimental group/average tumor weight of NC group) × 100%. During the treatment, no mice died from tumor loading. After 21 days of treatment, all animals were sacrificed by cervical dislocation at the termination of experiments, and the tumors were removed and weighed. Kidneys of DHA group were excised, fixed in 10% neutral-buffered formalin, and embedded in paraffin. Kidney sections (2 μm in thickness) were stained with hematoxylin-eosin (H&E) and Masson trichrome.

All animals were maintained in SPF facility with constant temperature (22-24°C) and a dark-light cycle of 12 h/12 h, and housed in plastic cages. The protocol was approved by the Ethics Committee for Animal Experiment of Bethune International Peace Hospital (Permit number: 20160058).

### Statistical analysis

All statistical tests were performed by SPSS19.0 statistics software (SPSS, Chicago, IL). All *in vitro* experiments were repeated at least three times. Data were presented as means ± SD. When more than two groups were enrolled, the means were compared between each two groups with one-way ANOVA or Student's t test. Differences with *P* < 0.05 were considered statistically significant.
